# Extensive Mitogenomic Remodeling Delineates the Family-Level Split in Velvet Worms

**DOI:** 10.3390/genes17040372

**Published:** 2026-03-25

**Authors:** Yaping Mi, Qunfei Guo, Pei Zhang, Youliang Pan, Wei Jiang, Wei Dai, Ying Wang, Shiwei Wang, Qiye Li

**Affiliations:** 1 Key Laboratory of Resource Biology and Biotechnology in Western China, Ministry of Education, Provincial Key Laboratory of Biotechnology, College of Life Sciences, Northwest University, 229 Taibai North Road, Xi’an 710069, China; miyaping@genomics.cn (Y.M.);; 2 BGI Research, Wuhan 430074, China; guoqunfei@genomics.cn (Q.G.); zhangpei@genomics.cn (P.Z.); panyouliang@genomics.cn (Y.P.);; 3 State Key Laboratory of Genome and Multi-Omics Technologies, BGI Research, Shenzhen 518083, China; jiangwei2@genomics.cn; 4 Shenzhen Key Laboratory of Forensics, BGI Research, Shenzhen 518083, China; 5 College of Life Sciences, University of Chinese Academy of Sciences, Beijing 100049, China

**Keywords:** Onychophora, mitochondrial genome, gene rearrangement, Panarthropoda, phylogeny, GC skew, Peripatidae, Peripatopsidae

## Abstract

Background: Velvet worms (Onychophora) occupy a pivotal phylogenetic position for deciphering the evolution of Panarthropoda, yet their exact placement within this clade remains debated. Furthermore, early studies in some onychophoran species revealed extensive gene rearrangements and the truncation or even loss of canonical transfer RNAs (tRNAs), features uncommon in other panarthropods. However, due to sparse representation, the pervasiveness and evolutionary significance of these genomic peculiarities across the phylum remain poorly understood. Methods: We sequenced and assembled three novel mitogenomes representing both extant onychophoran families (*Epiperipatus barbadensis* [Peripatidae]; *Euperipatoides rowelli* and *Phallocephale tallagandensis* [Peripatopsidae]) and conducted comparative analyses with five published species. Results: Onychophoran mitogenomes displayed high A+T content (mean 77.32%) but revealed a family-level divergence in GC skew. All genomes contained the standard 13 protein-coding genes (PCGs) and two ribosomal RNAs, yet tRNA counts varied significantly (ranging from 13 to 22), reflecting lineage-specific tRNA loss. Ancestral state reconstruction uncovered deep architectural divergence: Peripatopsidae retains the ancestral onychophoran gene arrangement, whereas Peripatidae exhibits a stable but derived gene order. Despite this architectural plasticity, synonymous codon usage patterns remained strictly conserved across the phylum, with all but one PCG evolving under strong purifying selection. Maximum likelihood phylogenetic reconstruction based on PCGs strongly supported Onychophora as the sister group to Arthropoda within Panarthropoda. Conclusions: Our findings provide robust molecular evidence supporting the Antennopoda hypothesis over the Tactopoda hypothesis for Panarthropoda phylogeny. Furthermore, we demonstrate extensive mitogenomic remodeling between the two extant onychophoran families, including divergent GC-skew patterns, tRNA contents, and gene arrangements.

## 1. Introduction

The evolution of Panarthropoda—the clade comprising Arthropoda, Tardigrada, and Onychophora—represents one of the most profound morphological transitions in animal history, spanning the shift from soft-bodied lobopodians to armored, jointed-limb arthropods [1]. Yet, the internal phylogeny of this clade remains controversial, centering on two competing hypotheses: Antennopoda, which posits Onychophora as the sister group to Arthropoda [2], and Tactopoda, which unites Tardigrada and Arthropoda [3]. Resolving this relationship is pivotal for reconstructing the origin and evolutionary sequence of key panarthropod innovations, such as complex brains, jointed appendages, and moulting exoskeletons.

In this context, velvet worms (Onychophora) occupy a unique position. Often characterized as “living fossils” [4], these cryptic, soil-dwelling invertebrates have retained a body plan remarkably similar to Cambrian lobopodians for over 300 million years [5]. This evolutionary stasis renders them indispensable for reconstructing ancestral traits and evolutionary history of panarthropods. Mitochondrial genomes (mitogenomes) have been widely demonstrated to offer high-resolution phylogenetic signal to resolve deep evolutionary relationships [6]. However, the realization of this potential is currently hindered by severe data paucity, with complete mitogenomes available for only five of the more than 200 known onychophoran species [7,8,9,10].

Furthermore, onychophoran mitogenomes exhibit exceptional features relative to other animals. Early studies reported the loss of up to nine of the 22 canonical mitochondrial transfer RNAs (tRNAs) in *Epiperipatus biolleyi* [8] and six in *Metaperipatus inae* [9]. In *Oroperipatus* sp. *DVL-2011*, and *Peripatoides sympatrica*, mitochondrial tRNA genes are typically truncated [10]. Through mitochondrial DNA and corresponding tRNA cDNA sequencing, it has been determined that extensive tRNA editing, sometimes involving the addition of up to 34 nucleotides, is required to restore the conventional structures of mature tRNAs and rebuild the aminoacyl acceptor stem [8]. Moreover, *M. inae* and *E. biolleyi* exhibit large-scale gene rearrangements that defy the conservation patterns typical of other ecdysozoans [9,10]. However, due to limited taxonomic sampling, the pervasiveness of these genomic peculiarities remains to be determined.

In this study, we assembled mitogenomes from three pivotal species across both extant onychophoran families. These include the viviparous *Epiperipatus barbadensis* (Peripatidae), one of the most common velvet worm species kept in captivity; the ovoviviparous *Euperipatoides rowelli* (Peripatopsidae), an emerging model organism for evolutionary biology; and *Phallocephale tallagandensis* (Peripatopsidae), the sole species of its monotypic genus. By integrating these new data with existing resources, we aim to (1) robustly resolve whether Onychophora or Tardigrada is the sister group to Arthropoda using expanded Onychophora mitogenomic data; (2) assess whether tRNA truncation or loss and gene rearrangements are intrinsic features of onychophorans; and (3) identify stable genomic features that distinguish the two extant families. Our results provide robust molecular evidence supporting the Antennopoda hypothesis, resolving the sister-group relationship between Onychophora and Arthropoda. Moreover, we uncover a complex divergence in mitogenomic architecture: while Peripatidae exhibit a highly stable but derived gene order, Peripatopsidae largely retain the ancestral arrangement yet display lineage-specific potential for extensive restructuring, as exemplified by *M. inae*. These findings advance our understanding of mitogenome evolution in Onychophora and establish a vital genomic framework for deciphering the deep phylogeny of Panarthropoda.

## 2. Materials and Methods

### 2.1. Sample Collection, DNA Extraction and Sequencing

Specimens of *E. barbadensis* were purchased from PG Exotic Pets Enterprise (Penang, Malaysia). Specimens of *E. rowelli* and *P. tallagandensis* were collected from New South Wales, Australia. All samples were frozen via liquid nitrogen and stored in a −80 °C freezer after collection. High-molecular-weight genomic DNA was extracted using the CTAB method as described by Guo et al. [11]. DNA concentration and integrity were evaluated by Qubit 3.0 (Invitrogen, Carlsbad, CA, USA) and Qsep 400 (BiOptic, Changzhou, China). Short-insert whole-genome sequencing (WGS) libraries were constructed using qualified DNA and sequenced on the DNBSEQ platform with the PE100 chemistry following the manufacturer’s instructions. Taxonomic identities of the specimens were validated by aligning the mitochondrial *COX1*, 12S rRNA, and 16S rRNA sequences derived from the assembled mitogenomes against corresponding sequences in NCBI GenBank.

### 2.2. Mitogenome Assembly and Annotation

Raw DNBSEQ WGS data was first filtered using SOAPnuke v1.5.6 [12] to remove adapters and low-quality reads (parameters *-Q 2 -G -d -l 20 -q 0.2 -5 1 -t 5,0,5,0*). This software was chosen for its high efficiency and comprehensive quality-control capabilities tailored to high-throughput sequencing data. The statistics of the raw and cleaned sequencing data are summarized in Supplementary Table S1. The cleaned data was then assembled using GetOrganelle v1.7.5 [13] (parameters: *-F animal_mt -R 15 -t 8 -k 21,45,65,85,105*), a toolkit that has been successfully employed in numerous recent metazoan mitogenome studies for *de novo* circular mitogenome assembly [14]. Specifically, a multi-scale k-mer strategy (corresponding to the parameter -k 21,45,65,85,105) was employed to construct the de Bruijn graph, with contigs iteratively expanded from the seed sequence for 15 rounds as specified by -R 15. The assembly process was guided by the built-in animal mitochondrial reference database designated by -F animal_mt.

Nucleotide skews of the mitogenomes were determined using the formulas: AT skew = (A − T)/(A + T) and GC skew = (G − C)/(G + C). Mitogenome annotation was performed using the MITOS2 web server [15] with default parameters to predict protein-coding genes (PCGs), tRNAs, and ribosomal RNAs (rRNAs). To improve the accuracy of tRNA identification, the candidate tRNA sequences initially annotated by MITOS2 were subjected to additional annotation validation with tRNAscan-SE v2.0.12 [16], followed by minimum free energy confirmation using RNAfold v2.7.0 [17]. To minimize missed detections, we also conducted a homology-based search using the complete tRNA set from *P. sympatrica* (which retains all 22 mitochondrial tRNAs) as queries with BLASTn (*-evalue 1e−3 -perc_identity 50 -word_size 7 -reward 1 -penalty −1 -gapopen 1 -gapextend 2*), followed by manual inspection of candidate regions for tRNA motifs. This combined approach ensured robust detection of both complete and truncated tRNA genes. The mitogenome maps were visualized using the CGView tool within Proksee (2023) [18].

### 2.3. Phylogenetic Analysis

To reconstruct the phylogeny of Panarthropoda and clarify the evolutionary position of Onychophora, we utilized 13 PCGs commonly found in metazoan mitogenomes. Mitogenomes of 18 representative species from Tardigrada and Arthropoda (Supplementary Table S2) were retrieved from GenBank, with the mollusk *Octopus bimaculoides* selected as the outgroup. Amino acid sequences of the 13 shared PCGs across the examined species were extracted and aligned using MAFFT v7.471 [19]. To generate codon-aware nucleotide alignments, the corresponding coding sequences (CDSs) were mapped from the protein sequence alignments using a custom Perl script, thereby strictly preserving the reading frames. These codon-aligned sequences of the 13 PCGs were subsequently concatenated into a single supermatrix. Finally, a Maximum Likelihood (ML) tree was constructed using IQTREE v3.0.1 [20] under the best-fit substation model selected by the ModelFinder (2017) [21]. Nodal support was assessed by 10,000 ultrafast bootstrap replicates. To reduce phylogenetic noise, the *--robust-phy* parameter was applied to retain the top 95% of sites with the highest likelihood values while excluding the lowest 5%. The final phylogenetic tree was visualized using iTOLs v6 [22].

### 2.4. Gene Rearrangement Analysis

Mitochondrial PCGs of the three newly assembled mitogenomes and five previously published ones (*Oroperipatus* sp. *DVL-2011*, *E. biolleyi*, *M. ina*, *Opisthopatus cinctipes*, and *P. sympatrica*) were used to analyze mitogenome rearrangement in Onychophora. Prior to analysis, all PCG sequences were verified for accuracy by comparing annotation results from MITOS2 with BLAST v2.11.0 searches against NCBI Genbank. To facilitate standardized comparison, the gene order of each species was re-oriented to start with *COX1*. Rearrangement events were identified by comparing the linearized gene orders of Onychophora species against the inferred ancestral gene order. The horseshoe crab *Limulus polyphemus* (Arthropoda) was chosen as the outgroup for reconstruction of ancestral gene order due to its well-preserved ancestral gene arrangement [23].

### 2.5. Codon Usage Bias Analysis

Mitochondrial codon usage was analyzed using CodonW v1.4.4 [24]. The CDSs of the 13 PCGs in the mitogenomes were imported into CodonW to calculate the Relative Synonymous Codon Usage (RSCU) for each codon. RSCU measures the usage bias of a specific codon relative to its synonymous counterparts. It is calculated by dividing the observed frequency of codon by its expected frequency under the assumption of equal usage, thereby eliminating the influence of amino acid composition and gene length. An RSCU of 1 indicates no bias; RSCU > 1 indicates that its usage frequency is higher than expected; and RSCU < 1 indicates that its usage frequency is lower than expected [25].

### 2.6. Amino Acid Substitution Rate Analysis

Multiple sequence alignment of the 13 shared PCGs (*ND6*, *ND4L*, *ND4*, *ND2*, *COX1*, *COX2*, *ATP8*, *ATP6*, *COX3*, *ND3*, *CYTB*, *ND1*, *ND5*) was performed using MAFFT v7.526 (parameters: *--anysymbol --maxiterate 1000 --localpair --quiet --thread 4*). Amino acid substitution rates for individual PCGs were subsequently calculated using the CODEML program in PAML based on the aligned amino acid sequences [26]. The analysis was configured for amino acid sequences (seq type = 2) with the empirical evolutionary model (model = 2) and the mitochondrial mtREV24 substitution matrix. The invertebrate mitochondrial genetic code was specified (icode = 4) and ambiguous alignment sites were excluded (clean data = 1). Rate heterogeneity across sites was modeled with a discrete gamma distribution (ncatG = 3, alpha = 0.5). All calculations were conducted without a molecular clock (clock = 0) using the phylogenetic tree described in 3.2, and amino acid substitution rates were derived from the CODEML output files.

## 3. Results

### 3.1. Features of the Newly Assembled Onychophoran Mitogenomes

**Figure 1:**
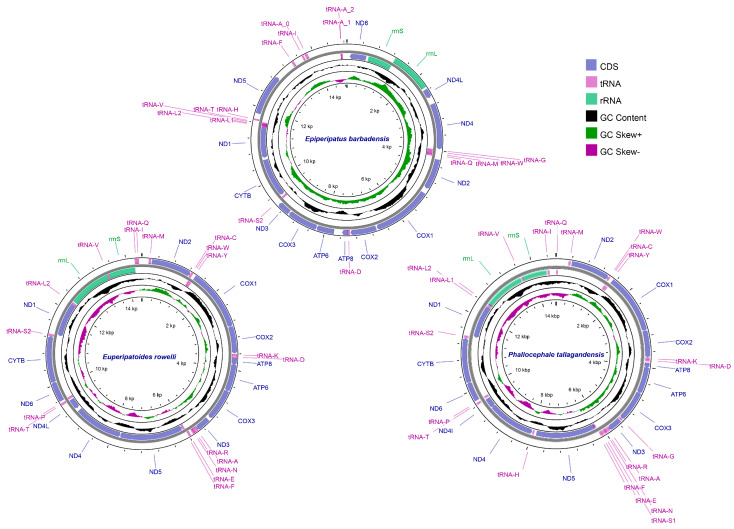
Visualization of the mitogenomes of the three Onychophora species. Colored blocks denote individual genes, with those on the outside and inner rings corresponding to genes encoded on the major strand and minor strand, respectively. Gene names are shown outside the rings.

To characterize the mitochondrial genomic landscape of Onychophora, we *de novo* assembled the mitogenomes of three species spanning its two extant families: *E. limulus* (Peripatidae), *E. rowelli* (Peripatopsidae) and *P. tallagandensis* (Peripatopsidae) (Figure 1; Supplementary Table S3). Taxonomic identities were verified by aligning their mitochondrial *COX1*, 12S rRNA, and 16S rRNA sequences against those available in public reference databases (Supplementary Table S4). The assembly of *E. barbadensis* was circularized, whereas those for *E. rowelli* and *P. tallagandensis* did not achieve circularization. Examination of read mapping to the assembled mitogenomes revealed that *E. barbadensis* exhibited relatively uniform coverage across its the entire mitogenome assembly, supporting a complete and accurate assembly. In contrast, the coverage profiles of *E. rowelli* and *P. tallagandensis* displayed elevated coverage at both the beginning and end of the linearized representation, suggesting the presence of highly similar repeat regions that hindered circularization (Supplementary Figure S1). Nevertheless, all three assemblies are highly complete, with sizes (14.4–14.9 kb) falling within the expected onychophoran mitogenome range (Supplementary Table S3).

Consistent with most metazoans, these three onychophoran mitogenomes encode the standard complement of 13 PCGs and rRNAs (Table 1 and Figure 1). However, the tRNA gene repertoire varies drastically across the three species, ranging from the complete canonical set of 22 tRNAs in *P. tallagandensis* to the reduced sets in *E. rowelli* (18 tRNAs) and *E. barbadensis* (14 tRNAs). Another notable divergence lies in the GC skew between *E. barbadensis* and the two Peripatopsidae species (*E. rowelli* and *P. tallagandensis*), which will be explored further in a later section.

### 3.2. Mitogenomes Resolve Onychophora as the Sister Group to Arthropoda

**Figure 2:**
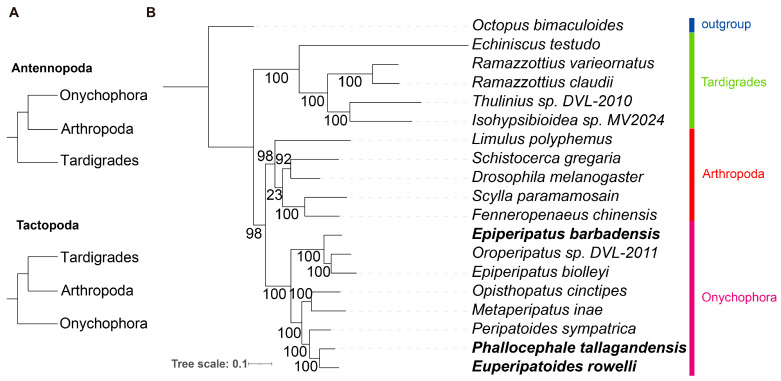
Mitogenomic phylogeny of Panarthropoda. (**A**) Two competing hypotheses for the internal phylogeny of Panarthropoda. (**B**) The Maximum-likelihood (ML) phylogenetic tree of Panarthropoda inferred from 13 mitochondrial PCGs. Bootstrap values are displayed at each node. Species names in bold denote the species sequenced in this study.

Mitochondrial PCGs encode core components of the oxidative phosphorylation system and are widely used for phylogenetic inference in the animal kingdom due to their conserved gene content and clearly orthologous relationships [27]. To resolve the phylogenetic position of Onychophora within Panarthropoda, we constructed a ML phylogenetic tree based on the 13 conserved mitochondrial PCGs across 18 panarthropod species and an outgroup species (*O. bimaculoides*) (Figure 2). The overall tree topology was highly robust, with 14 of 16 nodes receiving bootstrap support greater than 95% (including 12 nodes at 100%). Within the onychophoran lineage, the two extant families (Peripatidae and Peripatopsidae) were recovered as reciprocally monophyletic with 100% bootstrap support. Crucially, our analysis strongly supported Onychophora as the sister group to Arthropoda (98% bootstrap support), corroborating the Antennopoda hypothesis over the alternative Tactopoda hypothesis.

### 3.3. Mitochondrial Gene Rearrangements in Peripatidae and Peripatopsidae

**Figure 3:**
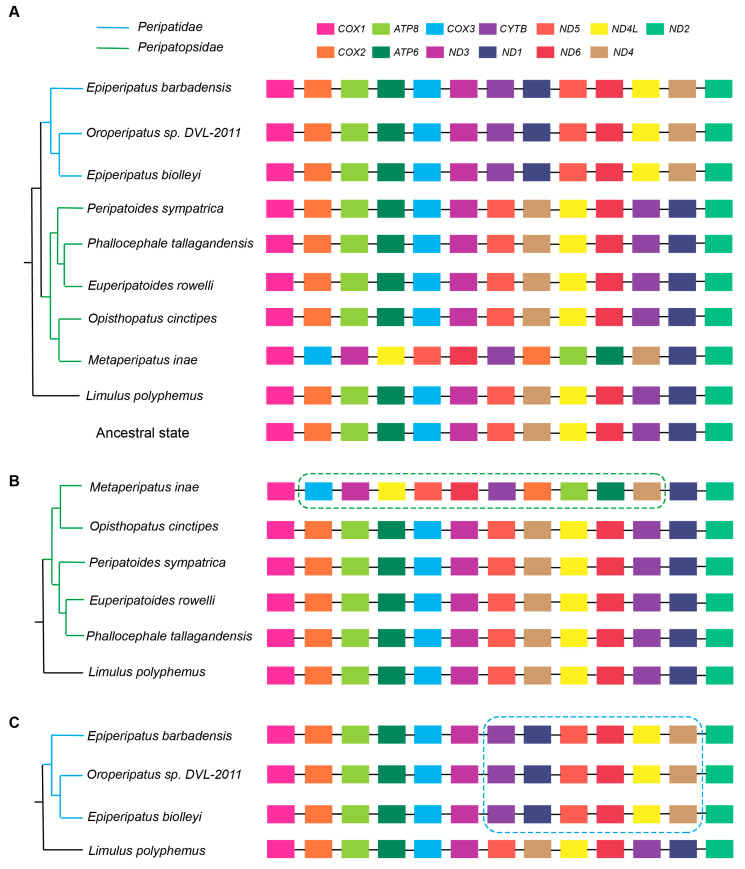
Phylogeny and mitochondrial gene order of Onychophora. (**A**) A maximum-likelihood tree (inferred from 13PCGs) of Onychophora, with branches colored by family (blue = Peripatidae; green = Peripatopsidae). To the right of each taxon, schematic mitochondrial gene arrangements are shown, with distinct colors representing individual genes. (**B**) Focus on the Peripatopsidae clade, with a dashed green box denoting the mitochondrial gene order regions that have been rearranged compared to the ancestral state (represented by *Limulus polyphemus*). The unique, rearranged gene order of *Metaperipatus inae* is presented as a contrast to the conserved gene arrangement pattern shared by most genera within this clade. (**C**) Focus on the Peripatidae clade, with a dashed blue box highlighting the region of mitochondrial gene order that has undergone rearrangement relative to the ancestral state (represented by *L. polyphemus*).

To trace mitochondrial gene rearrangement throughout the evolution of Onychophora, we reconstructed the ancestral gene order of the 13 PCGs using the horseshoe crab *L. polyphemus* (Arthropoda) as the outgroup. This revealed that last common ancestor of Onychophora maintained a gene order identical to that of *L. polyphemus* (Figure 3A). The Peripatopsidae lineage generally retains this ancestral arrangement of gene order, as exemplified by *O. cinctipes*, *E. rowelli*, *P. tallagandensis* and *P. sympatrica*, all of which exhibit identical gene order with *L. polyphemus* (Figure 3B). The sole exception among the examined Peripatopsidae species is *M. inae*, which exhibits extensive gene rearrangement, likely representing lineage-specific changes restricted to this genus.

In stark contrast, the Peripatidae lineage shows a gene arrangement diverges significantly from the ancestral pattern (Figure 3C). Notably, this novel arrangement is shared by all examined species within the family. This suggests that the evolutionary divergence of Peripatidae from Peripatopsidae was likely accompanied by substantial mitogenomic rearrangement.

### 3.4. Divergence in tRNA Content and Arrangement Between Peripatidae and Peripatopsidae

**Figure 4:**
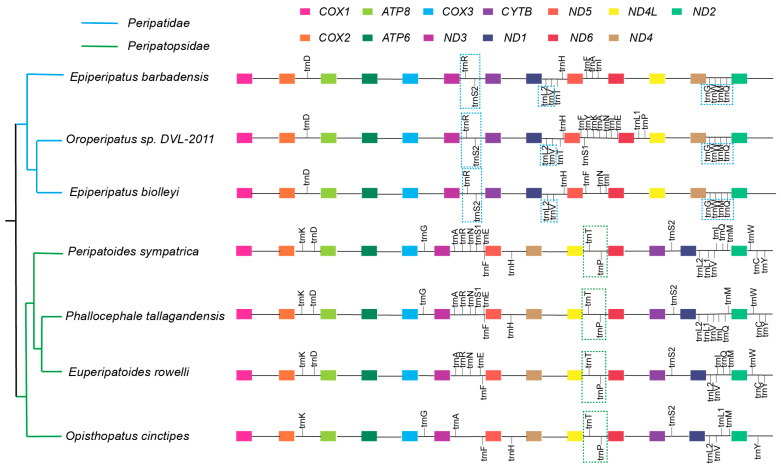
Mitochondrial tRNA distribution of Onychophora. The distribution of tRNA genes and their positional relationships with PCGs (colored blocks) in the mitogenomes of seven Onychophora species, grouped by family (Peripatidae: blue; Peripatopsidae: green). Vertical lines denote tRNA genes; tRNAs located above the connecting line are encoded on the major strand (positive strand), while those below the line are encoded on the minor strand (negative strand). Note: *M. inae* was not included in this figure because it exhibits a lineage-specific mitochondrial gene rearrangement that is highly divergent from the remaining taxa, as shown in Figure 3.

Consistent with most bilaterians, all eight examined Onychophora species contain a single copy of both the 16S and 12S rRNA genes—the two canonical ribosomal RNA components of metazoan mitochondrial translation [27]. However, we observed pronounced variation in mitochondrial tRNA gene content across species, ranging from 13 tRNA genes in *E. biolleyi* and *O. cinctipes* to 22 in *P. tallagandensis*, *M. inae* and *P. sympatrica* (Supplementary Table S5). Interestingly, Peripatopsidae species generally exhibit tRNA counts closer to the bilaterian standard. In contrast, Peripatidae species frequently show an apparent loss of tRNA genes, a pattern that aligns with the highly truncated and post-transcriptionally edited nature of tRNAs in this family [8]. Family divergence was also evident in tRNA arrangement: Peripatidae species shared stable tRNA gene clusters such as *trnR-trnS2*, *trnL2-trnV* and *trnG-trnW-trnM-trnQ*, whereas Peripatopsidae species displayed unique combinations such as *trnT-trnP* (Figure 4). This differential organization of tRNA blocks mirrors the rearrangement patterns observed in PCGs, reinforcing the conclusion that the divergence between Peripatidae and Peripatopsidae was accompanied by extensive structural remodeling of the mitogenome.

### 3.5. Onychophoran Mitogenomes Exhibit Derived and Lineage-Specific Shifts in Nucleotide Composition

Nucleotide composition analysis of the eight onychophoran mitogenomes revealed a pronounced A + T bias, with the overall A + T content ranging from 74.09% to 79.32% (mean: 77.32%), indicating that a strong mutational bias towards adenine and thymine is a conserved feature across the phylum. However, despite this shared overall A + T richness, we observed striking divergence in GC-skew patterns between the two extant onychophoran families. Specifically, species within Peripatidae exhibited predominantly positive GC-skew values across the mitogenome. In contrast, species within Peripatopsidae displayed a skew profile partitioned almost equally into positive and negative segments along the mitogenome. A notable exception was *M. inae*. This species possesses an extensively rearranged mitogenome, and its GC-skew distribution is more similar to the globally positive landscape of Peripatidae rather than its con-familial relatives. This suggests a potential link between mitogenomic rearrangements and shifts in nucleotide compositional bias.

**Figure 5:**
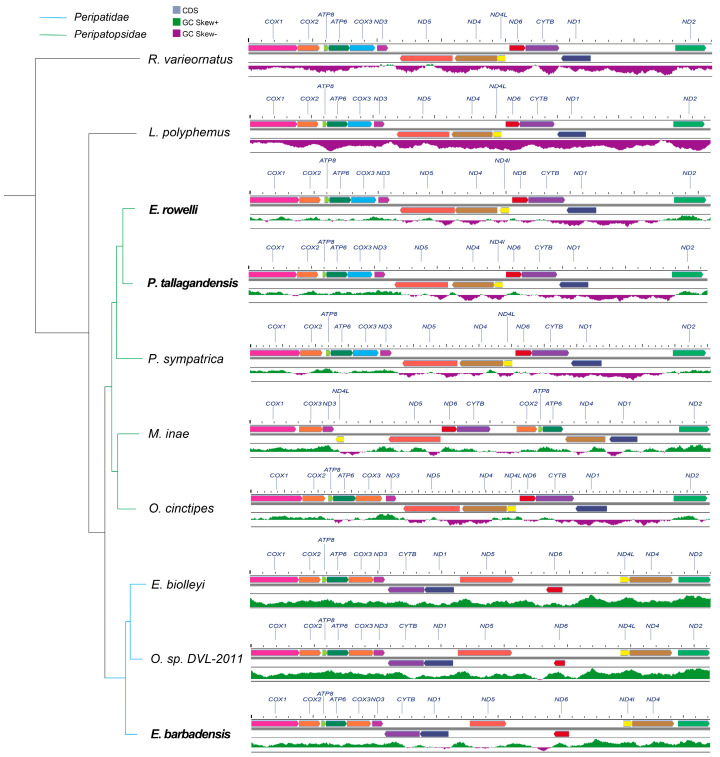
GC-skew profiles across the entire mitochondrial genomes. Left: phylogenetic relationships of examined species, with Peripatidae (blue) and Peripatopsidae (green) indicated. Right: linear mitochondrial genome maps for each species, showing protein-coding genes (colored boxes) and strand-specific GC-skew distributions (green/purple lines).

To track the origin of these compositional shifts, we evaluated the GC-skew landscapes in the sister panarthropod lineages, Arthropoda and Tardigrada. Species from both groups exhibited globally negative GC skews (Figure 5), indicating that the panarthropod ancestor possessed a mitogenome with an excess of cytosine over guanine on the coding strand. Consequently, the generally positive or bimodal GC-skew distributions observed in Onychophora represent derived states that emerged following its divergence from Arthropoda. We speculate that the ancestral onychophoran likely retained the state of a globally negative GC skew. During early diversification, the Peripatopsidae lineage underwent a partial compositional shift, resulting in the bimodal profile observed in most of its extant species. Conversely, the Peripatidae lineage underwent a more extreme evolutionary transition, completely shifting to a globally positive GC skew accompanied genome rearrangements.

### 3.6. Conserved Mitochondrial Codon Usage Across Panarthropoda

**Figure 6:**
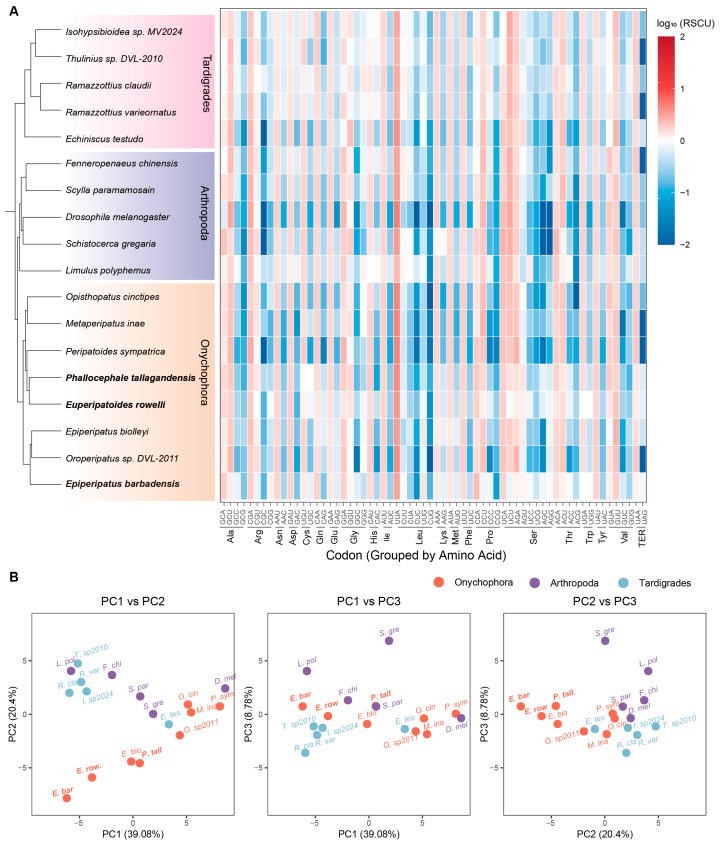
Comparative analysis of codon usage in panarthropod mitogenomes. (**A**) Relative Synonymous Codon Usage (RSCU) in eighteen panarthropoda mitogenomes, presented as log_10_ transformations. (**B**) Principal Component Analysis (PCA) score plot of Panarthropod species based on RSCU values.

Codon usage bias describes the preferential use of specific codons over their synonymous counterparts during translation [28]. We characterized codon usage patterns in mitochondrial PCGs across 18 panarthropod species using RSCU analysis (Supplementary Table S6). This revealed a strikingly similar codon usage profile across all species (Figure 6A), with codons terminating in A or U consistently yielding RSCU values > 1. This reflects strong mutational pressure toward A/T nucleotides at the neutrally evolving third codon position. In addition, principal component analysis (PCA) of the RSCU values demonstrated that species of Onychophora, Arthropoda, and Tardigrada exhibit an overlapping distribution within the multivariate space (Figure 6B). Together, these findings indicate that the foundational mutational pressures shaping panarthropod mitogenomes have remained relatively stable since the deep evolutionary divergence of these lineages.

### 3.7. Stringent Purifying Selection Constrains Onychophoran Mitogenomes

**Figure 7:**
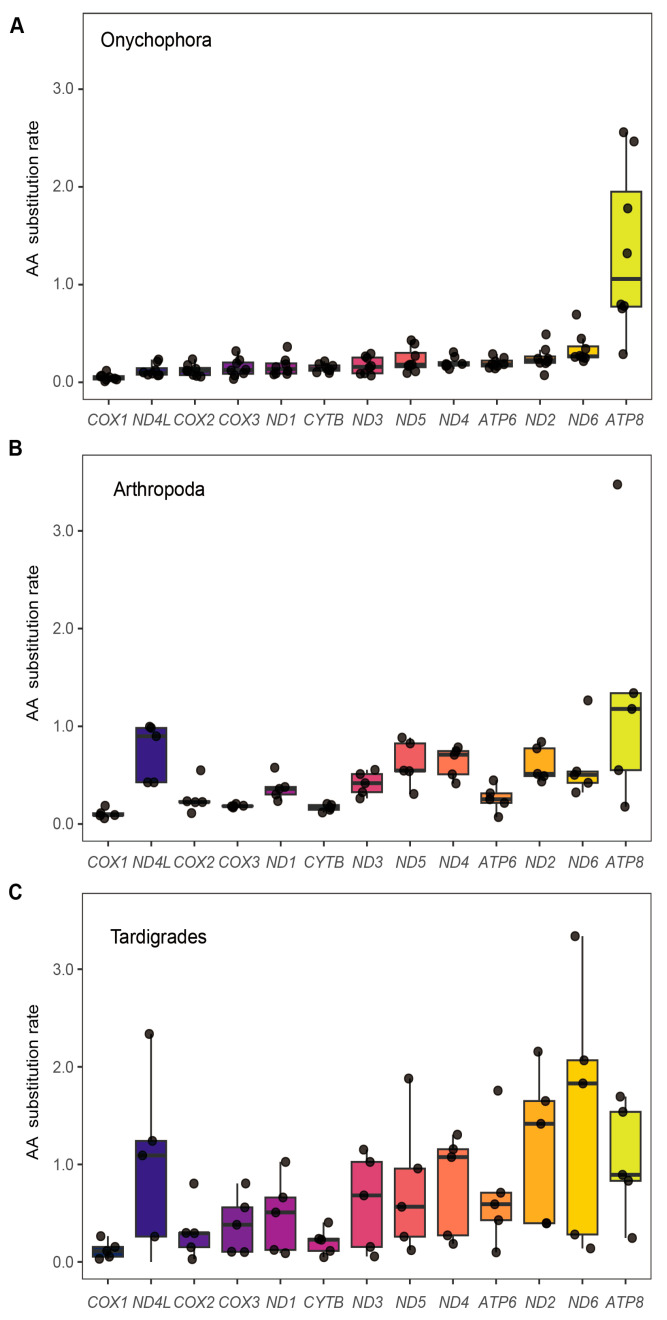
Boxplots showing amino acid (AA) substitution rates for the 13 mitochondrial protein-coding genes in three Panarthropoda lineages: (**A**) Onychophora, (**B**) Arthropoda, and (**C**) Tardigrades.

To evaluate the selective constraints acting on panarthropod mitogenomes, we analyzed amino acid substitution rates across the 13 PCGs for the three major lineages (Figure 7; Supplementary Table S7). In Arthropoda and Tardigrada, substitution rates varied substantially across genes. The cytochrome c oxidase subunits (*COX1*, *COX2*, *COX3*) and *CYTB* consistently exhibited the highest sequence conservation, whereas the NADH dehydrogenase subunits (*ND1*, *ND2*, *ND3*, *ND4*, *ND4L*, *ND5*, *ND6*) and *ATP8* demonstrated markedly elevated substitution rates. In stark contrast, nearly all PCGs in Onychophora displayed uniformly low substitution rates. The sole exception within this lineage was *ATP8*, which exhibited an anomalously high rate of sequence evolution. These patterns suggest that the onychophoran mitochondrial genes are generally subject to stringent purifying selection, with relaxed functional constraints acting specifically on this single ATP synthase subunit [29].

## 4. Discussion

By generating three new complete mitogenomes spanning both extant onychophoran families and integrating them with all previously available genomes, we substantially expand mitochondrial genome sampling for Onychophora—a phylum of key phylogenetic significance within Panarthropoda. Phylogenetic reconstruction based on this denser dataset robustly recovers Onychophora as the sister lineage to Arthropoda, supporting the Antennopoda hypothesis [23].

Notably, this expanded sampling reveals a previously unreported shift in GC-skew pattern that distinguishes the two onychophoran families from their panarthropod relatives. While arthropods and tardigrades exhibit globally negative GC skews, Onychophora displays lineage-specific derived states. Specifically, in Peripatidae, GC skew is consistently positive across the mitogenome, while Peripatopsidae generally displays a bimodal profile partitioned into positive and negative segments. Such pronounced family-level divergence in GC skew suggests altered strand-specific mutational pressures, a key driver of compositional asymmetry in metazoan mitogenomes [30,31]. Accordingly, the distinct GC-skew regimes between Peripatidae and Peripatopsidae likely reflect evolutionary changes in strand-specific mutation bias and differential selective constraints acting on mitochondrial protein-coding genes [32,33].

Overall, the two onychophoran families are separated by deep, family-level mitogenomic remodeling encompassing gene arrangements, tRNA complements and GC-skew landscapes. However, despite this structural and compositional divergence, synonymous codon usage remains strikingly conserved across Panarthropoda. Codon preferences in Onychophora align closely with those of arthropods and tardigrades, with consistent A/U bias at third codon positions. In addition, most onychophoran PCGs evolve under strong purifying selection with *ATP8* as a notable outlier, likely due to lineage-specific environmental adaptations [34,35].

Collectively, our findings establish Onychophora as an exceptional system for studying mitochondrial genome evolution, where profound structural reorganization and strand-specific compositional shifts coexist alongside deeply conserved codon usage and stringent functional constraints on most protein-coding genes [36,37].

## Figures and Tables

**Table 1:** Mitogenome assembly statistics for the three Onychophora species.

Species	Length (bp)	GC%	AT Skew	GC Skew	PCGs	tRNA	rRNA
*Epiperipatus barbadensis*	14,871	22.00	−0.06	0.15	13	14	2
*Euperipatoides rowelli*	14,386	22.79	−0.03	−0.01	13	18	2
*Phallocephale tallagandensis*	14,611	21.70	0.028	0.01	13	22	2

## Data Availability

DNBSEQ short-read WGS data is deposited in the CNGB Nucleotide Sequence Archive (CNSA) of China National Gene Bank DataBase (CNGBdb) under accession no. CNP0009099.

## Supplementary Materials

The following supporting information can be downloaded at: https://www.mdpi.com/article/10.3390/genes17040372/s1. Supplementary Table S1. The summary statistics of sequencing data generated for mitogenome assembly and annotation. Supplementary Table S2. All species information and mitochondrial genome accession numbers used for phylogeny analysis. Supplementary Table S3. The basic features of three mitochondrial genomes assembled in this study and five published genomes of Onychophora. Supplementary Table S4. Identity of mitochondrial PCGs between the study species and published Onychophora. Supplementary Table S5. Characterization of tRNA Genes in Eight Onychophora. Supplementary Table S6. Relative Synonymous Codon Usage of eighteen Panarthropoda species. Supplementary Table S7. Amino acid substitution rate of eighteen Panarthropoda species. Supplementary Figure S1. Sequencing depth coverage across three onychophoran mitochondrial genomes. The orange-filled plots show the read depth distribution across the entire mitogenome of *Epiperipatus barbadensis*, *Euperipatoides rowelli*, and *Phallocephale tallagandensis*. The red dashed line indicates the mean sequencing depth for each species.

## Abbreviations

The following abbreviations are used in this manuscript:

Mitogenomes	mitochondrial genomes
PCGs	protein-coding genes
rRNAs	ribosomal RNAs
tRNAs	transfer RNAs
CTAB	cetyl trimethylammonium bromide method
PFGE	pulsed field gel electrophoresis
CDS	coding sequences
RSCU	Relative Synonymous Codon Usage
ML	maximum likelihood
ATP6	ATP synthase 6
ATP8	ATP synthase 8
COX1	Cytochrome c oxidase I
COX2	Cytochrome c oxidase II
COX3	Cytochrome c oxidase III
CYTB	Cytochrome b
ND1	NADH dehydrogenase 1
ND2	NADH dehydrogenase 2
ND3	NADH dehydrogenase 3
ND4	NADH dehydrogenase 4
ND4L	NADH dehydrogenase 4L
ND5	NADH dehydrogenase 5
ND6	NADH dehydrogenase 6
