# Supplementary material for: Extensive Mitogenomic Remodeling Delineates the Family-Level Split in Velvet Worms
Source: Genes (Basel). 2026 Mar 25;17(4):372. doi: 10.3390/genes17040372 (PMC13116273; doi:10.3390/genes17040372)
Supplement: Supplementary file 1 [file genes-17-00372-s001.zip › genes-4213463-supplementary figure.pdf]

genes

MDPI

Supplementary Materials

Extensive Mitogenomic Remodeling Delineates the Family-Level Split in Velvet Worms

Yaping Mi [1,2], Qunfei Guo [2], Pei Zhang [2,3], Youliang Pan [2], Wei Jiang [3], Wei Dai [2], Ying Wang [2], Shiwei Wang [1] and Qiye Li [3,4,5,*]

1 Key Laboratory of Resources Biology and Biotechnology in Western China, Ministry of Education, Provincial Key Laboratory of Biotechnology of Shanxi Province, The College of Life Sciences, Northwest University, Xi'an 710069, China; miyaping@genomics.cn (Y.M.)
2 BGI Research, Wuhan 430074, China; guoqunfei@genomics.cn (Q.G.); zhangpei@genomics.cn (P.Z.); panyouliang@genomics.cn (Y.P.)
3 State Key Laboratory of Genome and Multi-Omics Technologies, BGI Research, Shenzhen 518083, China; jiangwei2@genomics.cn
4 Shenzhen Key Laboratory of Forensics, BGI Research, Shenzhen 518083, China
5 College of Life Sciences, University of Chinese Academy of Sciences, Beijing 100049, China
* Correspondence: liqiye@genomics.cn

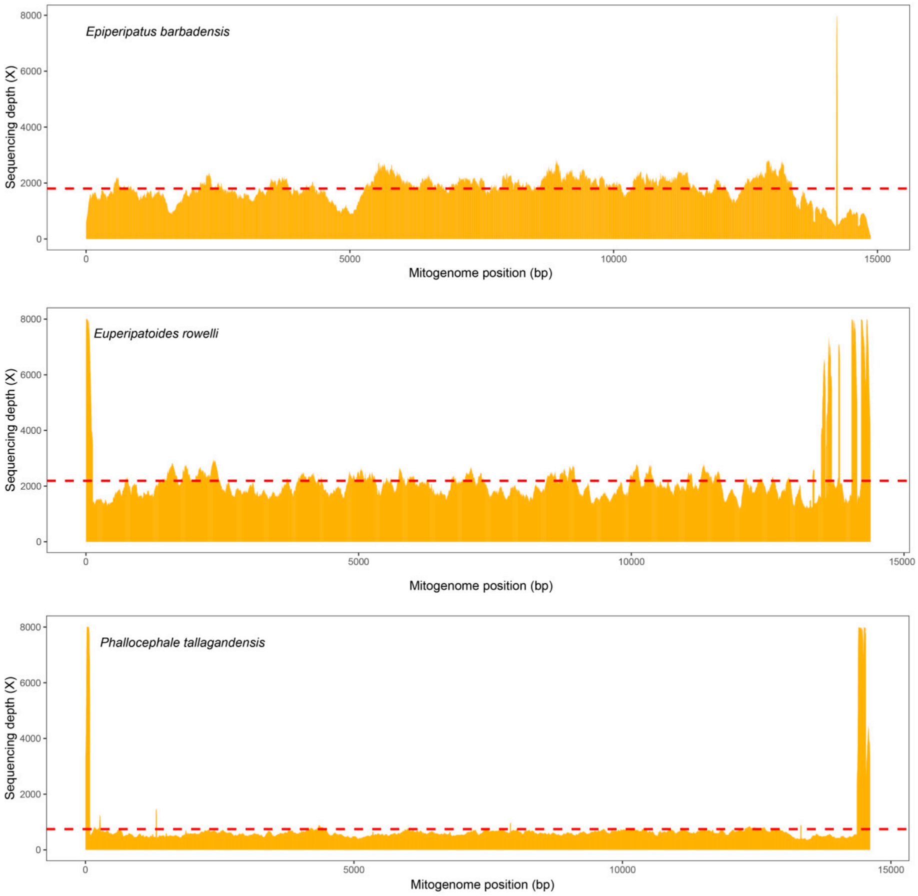

Supplementary Figure S1. Sequencing depth coverage across three onychophoran mitochondrial genomes. The orange-filled plots show the read depth distribution across the entire mitogenome of *Epiperipatus barbadensis*, *Euperipatoides rowelli*, and *Phallocephale tallagandensis*. The red dashed line indicates the mean sequencing depth for each species.
